# Vaccinia Virus in Household Environment during Bovine Vaccinia Outbreak, Brazil

**DOI:** 10.3201/eid1912.120937

**Published:** 2013-12

**Authors:** Felipe L. Assis, Iara A. Borges, Vaz S. Mesquita, Paulo C. Ferreira, Giliane S. Trindade, Erna G. Kroon, Jonatas S. Abrahão

**Affiliations:** Universidade Federal de Minas Gerais, Belo Horizonte, Minas Gerais, Brazil (F.L. Assis, I.A. Borges, P.C. Ferreira, G.S. Trindale, E.G. Kroon, J.S. Abrahão);; Serviço Médico Municipal, Carangola, Minas Gerais, Brazil (V.S. Mesquita)

**Keywords:** vaccinia virus, orthopoxvirus, viruses, outbreak, poxvirus, bovine vaccinia, household environment, Brazil

**To the Editor**: Several exanthematic vaccinia virus (VACV) outbreaks have affected dairy cattle and rural workers in Brazil and Asia, and have caused economic losses and affected health services (*1**–**3*). VACV, the prototype of the genus *Orthopoxvirus* (OPV), exhibits serologic cross-reactivity with other OPV species and was used during the smallpox eradication campaign (*1*). Several VACV strains have been isolated during bovine vaccinia outbreaks in Brazil and have been characterized by molecular and biologic methods (*3**,**4*). Bovine vaccinia infections in humans are frequently related to occupational contact with sick animals during milking but have never been shown to be associated with fomites or indoor environments (*1**,**3*). 

In August 2011, a bovine vaccinia outbreak was reported in Carangola County, Minas Gerais State, Brazil. During this outbreak, several farms were affected, and the outbreak involved humans and dairy cattle. A 41-year-old man (patient 1) who worked on a farm (20°36′30.7′′S, 42°17′53.9′′W) was hospitalized. He had painful lesions on the hands, high fever, lymphadenopathy, malaise, and fainting episodes. This patient reported recent contact with sick animals on the farm during milking.

At the same time, a 57-year-old man (patient 2), the owner of the farm, had a lesion on the right hand. This infection was also related to occupational exposure. Some days after the appearance of the hand lesion, this patient presumably inoculated himself at the site of an abrasion he had recently received on his nose. This resulted in development of a large and painful lesion. This patient reported milking cows daily. He had been vaccinated against smallpox before 1977.

A total of 5 humans and 15 cows were involved in this outbreak on 5 farms. Clinical samples were obtained from the 2 patients and from 3 sick cows. Dried swab specimens from lesions were soaked in 200 μL of phosphate-buffered saline containing penicillin (200 U/mL), amphotericin B (4 μg/mL), and gentamicin (100 μg/mL); homogenized, and centrifuged at 2,000 × *g* for 3 min. The supernatants were used for molecular diagnosis and virus isolation (*3**,**5**,**6*). Supernatants were tested by using OPV-specific PCRs that targeted the C11R gene, which encodes viral growth factor (*vgf*), and the A26L gene, which encodes A-type inclusion (ATI) protein. Samples from the 2 patients were positive for *vgf* and ATI (*7*). At least 1 sample (blood or scabs) from each sick animal was also positive by PCR.

To assess the risk for virus spread in indoor environments, we collected swab specimens from several objects, including doorknobs, bathroom surfaces, and the pillow of patient 2. The pillow was positive for *vgf* and ATI by PCR.

To isolate the virus, we infected monolayers of BSC-40 cells cultured in a 6-well plate with sample supernatants and incubated the cells at 37°C for 72 h or until a cytopathic effect was detected (*3**,**5**,**6*). We isolated virus from a sample from patient 1 and from an environmental sample (the pillow of patient 2), which showed positive results in the molecular diagnostic assays. 

To confirm that the isolated VACV was the OPV involved in this outbreak, we sequenced partial fragments of the A56R and A26L genes from the isolated virus. Fragments obtained were directly sequenced in both orientations in triplicate (MegaBACE 1000 Sequencer; GE Healthcare, Little Chalfont, UK). Sequences were aligned with published OPV sequences in GenBank by using the ClustalW (www.clustal.org/) method and manually aligned by using MEGA version 4.0 (Arizona State University, Phoenix, AZ, USA). VACV molecular signatures of 18-nt and 12-nt deletions were observed in the A56R and A26L genes, respectively. Phylogenetic trees (Figure), which were constructed by using the neighbor-joining method, the Tamura-Nei model of nucleotide substitutions, and 1,000 bootstrap replicates in MEGA 4.0, demonstrated that this isolate clustered with other group 1 VACV isolates from Brazil. We named this isolate Carangola virus.

**Figure:**
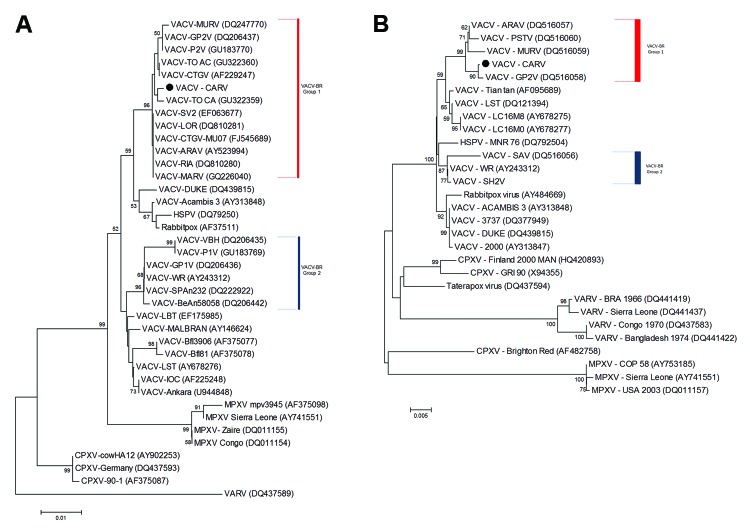
Phylogenetic trees based on orthopoxvirus nucleotide sequences, including vaccinia virus (VACV) from Brazil (VACV-BR). Phylogenetic analysis was performed for A56R (A) and A26L (B) gene sequences and grouped VACV-BR strains into 2 branches: group 1 (red bar) and 2 (blue bar). The Carangola virus (CARV) isolate is indicated by the black dots. Both trees show grouping of CARV into VACV-BR cluster composed of Guarani P2 virus (GP2V), Cantagalo virus (CTGV), and other viruses. Trees were constructed by using the neighbor-joining method, the Tamura-Nei model of nucleotide substitutions, and bootstrap values of 1,000 replicates in MEGA version 4.0 (Arizona State University, Phoenix, AZ, USA). GenBank accession numbers are indicated in parentheses. Values along the branches indicate bootstrap values. Scale bars indicate nucleotide substitutions per site. MURV, Muriaé virus; MARV, Mariana virus; HSPV, horsepox virus; MPXV, monkeypox virus; ARAV, Araçatuba virus; PSTV, Passatempo virus; VARV, variola virus.

We isolated VACV from an indoor environment during a bovine vaccinia outbreak. VACV infections have been frequently associated with occupational activities, primarily direct contact with sick animals (*1**,**3*). However, in some cases, the source of the infection is unknown, especially in patients who did not participate in milking activities.

Human-to-human transmission has been suggested to have occurred in some bovine vaccinia outbreaks in Brazil, and nosocomial infection has been reported Asia (*2**,**8*). Household transmission of VACV has also been described in the United States after contact with lesions of a smallpox vaccinee in the military (*9*). VACV from Brazil shows long-lasting stability under environmental conditions, especially when associated with organic matter (*10*). Although the wife of patient 2 did not exhibit any typical clinical symptoms of VAVC infection, we believe that relatives sharing household environments with patients with lesions may be at risk for VACV infection. Isolation of VACV from a household environment raises new questions about nonoccupational risk factors related to bovine vaccinia transmission.
